# Emotional Processing in Individuals with Substance Use Disorder and Posttraumatic Stress Disorder

**DOI:** 10.1007/s11469-016-9727-6

**Published:** 2017-01-12

**Authors:** Laura K. Kemmis, Shamil Wanigaratne, Kimberly A. Ehntholt

**Affiliations:** 1 Traumatic Stress Clinic, Camden & Islington NHS Foundation Trust, 4th Floor West Wing, St Pancras Hospital, St Pancras Way, London, NW1 0PE UK; 2 Institute of Psychiatry, Psychology and Neuroscience, King’s College London, 16 De Crespigny Park, London, SE5 8AF UK; 3 National Rehabilitation Centre, 55001, Abu Dhabi, United Arab Emirates

**Keywords:** Posttraumatic stress disorder (PTSD), Substance use disorder (SUD), Co-morbidity, Emotional processing, Treatment

## Abstract

Previous research has shown that individuals with substance use disorder (SUD) and posttraumatic stress disorder (PTSD) have emotional processing difficulties. However, no studies have specifically investigated the role of emotional processing in those with co-morbid SUD-PTSD. This study investigated whether there are more emotional processing abnormalities among patients with SUD-PTSD, than those with either a single diagnosis of PTSD or SUD. Emotional processing was assessed in three groups [1) SUD (without PTSD); 2) PTSD (without SUD); and 3) co-morbid SUD-PTSD] using the Emotional Processing Scale (EPS-25) and the International Affective Picture System (IAPS). Each of the three groups reported evidence of emotional processing dysfunction relative to the normal population. Within the SUD-PTSD group there was significant evidence that the additional impact of trauma increased emotional processing dysfunction but less evidence to suggest that substance use increased emotional processing dysfunction further. These findings call into question current United Kingdom guidelines for the treatment of co-morbid SUD-PTSD, which recommend that the drug or alcohol problem should be treated first.

Posttraumatic stress disorder (PTSD) and substance use disorder (SUD) have high co-morbidity (Kessler et al. 1995; Reynolds et al. 2004) and present a challenge for services. Patients with SUD-PTSD are typically more difficult to treat than those with either disorder alone (Shafer and Najavits 2007) and often have a higher prevalence of co-morbidity, suicidal ideation, poorer adherence to treatment (Back et al. 2009; Mills et al. 2006) and relapse more quickly (McCauley et al. 2012). Ongoing controversy exists in both the trauma and addiction fields regarding which disorder to treat first. In the USA, an integrated exposure-based treatment model has been advanced for SUD-PTSD (Najavits et al. 2005; see also Coffey et al. 2006; Mills et al. 2012). However, the two disorders are not usually treated simultaneously within the UK (NICE 2005) and trauma-focused exposure-based therapies remain largely underutilised with SUD-PTSD patients (Becker et al. 2004). Within the literature there appear to be two key barriers for providing integrated treatment for SUD-PTSD, especially the use of trauma-focused treatment and the application of exposure-based techniques. One barrier is that prolonged exposure (PE) treatment was traditionally considered inappropriate for use among patients with SUD due to safety concerns and risks of exacerbating symptoms (Foa and Rothbaum 1998). Another potential barrier is that *“substance (ab)use may prevent emotional processing”* and the exposure treatment would be less effective and *“may also lead to an exacerbation of PTSD symptoms”* (Ehlers and Steil 1995).

A recent review of the literature examined evidence from PE based RCT trials across common comorbid conditions including SUD-PTSD (van Minnen et al. 2015). Although the research is limited due to the exclusion of patients with substance dependence from most exposure based RCTs (Van Minnen et al. 2012), the evidence to date suggests that PE did not lead to an exacerbation of substance use of severity of SUD. In relation to the second rationale for not offering exposure based treatment, due to anticipated emotional processing problems, there is even less research. Previous research has shown that individuals with substance use disorder (SUD) and posttraumatic stress disorder (PTSD) have emotional processing difficulties. However, no studies to date have specifically explored the role of emotional processing in those with co-morbid SUD-PTSD. This study therefore investigates whether there are more emotional processing abnormalities among patients with SUD-PTSD, than those with either a single diagnosis of PTSD or SUD.

Four main pathways have been proposed for the high level of SUD-PTSD co-morbidity including the: i) self-medication hypothesis; (ii) high-risk hypothesis; iii) exacerbation and susceptibility hypotheses and iv) an indirect pathway. In line with the self-medication hypothesis (Khantzian 1985), Stewart and Conrod (2003) propose that PTSD can lead to SUD as substance use may function to regulate the negative emotion associated with PTSD symptoms. This is supported by studies that have found SUD patients with PTSD report trauma memories becoming more frequent and vivid after ceasing drug use and connect drug use with escape, blocking, numbing or helping them to cope (Reynolds et al. 2004). Other researchers have proposed the ‘high-risk’ hypothesis whereby the substance use leads to an increased risk of trauma exposure resulting in an increased risk of developing PTSD following trauma exposure (McFarlane 1998). Stewart (1996) proposes a further pathway, whereby once PTSD has developed, substance abuse could actually exacerbate or increase susceptibility of PTSD symptoms. Such patients may have emotional regulation problems including difficulties tolerating stress as well as high arousal states that motivate continued substance use (Stewart 1996; see also Hien et al. 2002). Finally, other researchers have explored the role of genetic, environmental and neurophysiological systems that may precede both SUD and PTSD or develop as a result of exposure to traumatic events (McCauley et al. 2012; McLeod et al. 2001).

As discussed, ongoing controversy exists in both the trauma and addiction fields regarding which disorder to treat first and two key concerns have been raised around offering trauma-focused treatment with SUD-PTSD patients that include PE. One treatment barrier relates to patient safety and risks of exacerbating symptoms (Foa and Rothbaum 1998) and the other concerns are that *“substance (ab)use may prevent emotional processing”* (Ehlers and Steil 1995).

In relation to patient safety, Najavits et al. (1998) put forward a treatment manual “Seeking Safety” that addressed key concerns around the safety of offering treatment for co-morbid SUD-PTSD. The original manual and associated RCTs did not include trauma-focused components such as exposure however, prioritised coping skills, stabilisation and establishing the patients’ sense of safety by working with both disorders simultaneously rather than trying to treat the disorders separately. Najavits (2002) acknowledged that eliciting trauma memories too early in treatment, when safety has not been established, may have harmful consequences ((Chu 1998; Ruzek et al. 1998) in Najavits 2002). However, by helping patients move toward safety, Najavits (2002) highlighted that the therapists can protect both themselves and their clients from any potential exacerbation of symptoms due to substance use or possible substance withdrawal, by supporting clients to identify alternative coping strategies early on in their treatment. The “Seeking Safety” manual was subsequently adapted to “Seeking Safety plus Exposure Therapy-Revised” which involved a combination treatment that included the former promotion of establishing safety through developing coping skills as well as exposure therapy, modified for PTSD and SUD.

There is therefore increasing evidence advocating for an integrated approach to the treatment of SUD-PTSD which includes prolonged exposure (Brady et al. 2001; Coffey et al. 2006; Mills et al. 2012; Najavits et al. 2005; Triffleman 2000;) as well as female methadone patients (Schiff et al., 2015). In a review of the literature on RCTs for the integrated treatment of SUD-PTSD, Gulliver and Steffen (2010) examined the controversy regarding best practices for psychotherapy, especially the application of exposure-based techniques and reported positive outcome data from RCTs and made recommendations for the integrated treatment of SUD-PTSD incorporating exposure. A recent review of the literature of exposure based RCT trials for SUD-PTSD also found that *“prolonged exposure did not lead to an exacerbation of substance use of severity of SUD”* (van Minnen et al. 2015).

There has been less research investigating the second barrier to offering exposure-based treatment due to concerns that, *“substance (ab)use prevents emotional processing and may exacerbate symptoms of PTSD”* (Ehlers and Steil 1995). It is postulated that support for this hypothesis can be demonstrated by findings that individuals with high alcohol consumption are at increased risk of developing PTSD after traumatic events (Feinstein and Dolan 1991, cited by Ehlers and Steil 1995). However although this study provides some evidence of PTSD symptom exacerbation for individuals who are intoxicated and presenting to accident and emergency services for physical trauma, it doesn’t actually provide any measure of emotional processing.

While the concept of emotional processing has clinical utility, its operational measurement may be problematic (Baker et al. 2007a). The concept of ‘emotional processing’ was originally defined by Rachman in the 1980s as: *“a process whereby emotional disturbances are absorbed, and decline to the extent that other experiences and behaviour can proceed without disruption”* (Rachman 1980 p. 51). Rachman later revised and applied the concept to PTSD (Rachman 2001). Emotional processing has been well documented in relation to PTSD and is central to all main theoretical and treatment models of PTSD (Foa and Kosak 1986; Foa and Riggs 1993; Foa and Rothbaum 1998; see also Brewin et al. 1996; Ehlers and Clark 2000). Emotional numbing is an indication of impaired emotional processing and is a critical symptom of PTSD (Foa et al. 1995; see also Reynolds et al. 2004). Abnormalities in emotional processing are also discussed within the addiction literature (e.g. Verdejo-García et al. 2006; Verdejo-García et al. 2007). These abnormalities have been associated with alterations in the limbic system as well as the orbitofrontal cortex (OFC) in lesion and imaging studies (Dom et al. 2005).

Several researchers have highlighted however, that whilst the concept of emotional processing appears clinically useful and relevant, research has been impeded by the lack of any psychometrically sound assessment instrument that encompasses the different facets of emotional processing (Baker et al. 2007a). The majority of researchers who have endeavoured to investigate emotional processing dysfunction use the “Looking at Pictures” test (Lang et al. 1993) in which subjects rate a series of pictures from the International Affective Picture System (IAPS). Responses to these images have emphasised two dimensions of emotion: “valence” (i.e. pleasantness) and “arousal” (i.e. activation), on the basis of a 2-factor model developed originally by Russell and Mehrabian (1977) and advanced by Lang et al. (1993; 1998). This methodology facilitates the assessment of two forms of emotional processing abnormalities: heightened negativity and emotional numbing, which can be further categorised into two types of numbing, ‘positive blunting’ and ‘negative blunting’. Positive blunting is the more characteristic of emotional numbing as defined by the DSM-IV criteria of PTSD (APA 1994).

At least four studies have explored emotional processing in PTSD using the International Affective Picture System (IAPS) task to evaluate evidence for two forms of emotional abnormality: numbing and heightened negative emotionality (Amdur et al. 2000; Litz et al. 2000; Spahic-Mihajlovic et al. 2005; Wolf et al. 2009) with conflicting results. Disparity in findings among this research could be due to the differences in trauma type, time elapsed since the trauma, or reflect a lack of clinical sensitivity and clinical significance for the IAPS. There are also mixed findings for the IAPS task in individuals with SUD and emotional processing abnormalities, with evidence of emotional numbing (Aguilar de Arcos et al. 2005), heightened negativity to unpleasant stimuli (Verdejo-García et al. 2006), evidence of emotional numbing and heightened negativity (Aguilar de Arcos et al. 2008) and no evidence of emotional numbing on the IAPS task in heavy cannabis users but evidence of abnormal medial prefrontal cortex activity during the emotional evaluation (Wesley et al. 2016). These differences appear to be influenced by dependence on certain drug types (e.g. stimulant versus depressant and acute versus chronic drug use).

Recent research has questioned the sensitivity of the IAPS rating protocol for the assessment of PTSD-related emotional numbing (Wolf et al. 2009). Several recommendations have been made to increase the sensitivity of the assessment tool including, increasing the number of pictures and including trauma-related images (Wolf et al. 2009). Baker et al. (2007b) have also reported on the development and preliminary psychometric evaluation of an Emotional Processing Scale (EPS). The EPS-25 contains 25 items and yields five factors of emotional processing including: (1) impoverishing emotional experience; (2) signs of unprocessed emotion; (3) avoidance; (4) suppression and (5) unregulated emotion (Baker et al. 2007b) (Fig. 1).

This study examines whether individuals with SUD-PTSD have more emotional processing abnormalities compared to individuals with PTSD alone and individuals with SUD alone. This study employs the IAPS emotional processing task applying a larger data set than in previous studies following recommendations to improve the sensitivity of the tool (Wolf et al. 2009). The study also employs the recently developed EPS-25 emotional processing scale. The nature of any reported emotional processing abnormalities are clarified and the impact on treatment outcome considered.

## Design

The study employed a between group design. Emotional processing was assessed in three groups: (1) individuals with SUD (without PTSD); (2) individuals with PTSD (without SUD); and (3) individuals with co-morbid SUD-PTSD.

## Method

### Participants

A total of 90 individuals, aged between 18 and 70 years, were recruited through a PTSD outpatient treatment service and inpatient and community addiction services. Clinicians identified suitable participants from their caseloads if they fulfilled criteria for either a diagnosis of SUD, PTSD or co-morbid SUD-PTSD and were in active treatment. Participants were required to have competency in spoken English in order to complete questionnaire measures. Participants were also asked to refrain from using illicit substances or alcohol within the past 24 h prior to attending the assessment. Three screening measures were employed including the Traumatic Stress Questionnaire (TSQ; Brewin et al. 2002) as well as the Alcohol Use Disorders Identification Test (AUDIT, Piccinelli et al. 1997) or the Severity of Dependence Scale (SDS, Gossop et al. 1995), depending on their substance of choice. Severity of PTSD and SUD symptom severity were assessed further during the follow up assessment using the Post-traumatic Stress Diagnostic Scale; PDS (Foa et al. 1997) and the Maudsley Addiction Profile: MAP (Marsden et al. 1998).

Of the 90 participants who completed the screening assessment, seven participants were excluded from the study for various reasons including intoxication at the follow up assessment. A total of eighty-three participants completed the study. Twenty-one were recruited through the PTSD treatment service for the PTSD-group. Twenty-six were recruited through addiction services for the SUD-group. Thirty-six participants were recruited from both services for the SUD-PTSD group. Only two participants who were recruited through the PTSD treatment service were receiving parallel treatment for their substance misuse through a community addiction service. No participants within the SUD-PTSD group were receiving integrated treatment for both disorders.

### Measures

#### Screening Measures

A semi-structured interview was conducted which included screening questions related to drug use and the experience of trauma, taken from the Psychiatric Diagnostic Screening Questionnaire (PDSQ; Zimmerman and Mattia 2001).

The Severity of Dependence Scale (SDS; Gossop et al. 1995) was used as a brief screening test to determine dependence on their primary drug of choice. The total score of the SDS items have been found to correlate with the SODQ as a measure of opiate dependence (Sutherland et al. 1986 in Gossop et al. 1995). Other studies have reported the measure’s sensitivity and specificity for detecting dependency of other substances including alcohol (Lawrinson et al. 2007), cannabis (Martin et al. 2006) and benzodiazepines (de las Cuevas et al. 2000).

The Alcohol Use Disorders Identification Test (AUDIT; Piccinelli et al. 1997) assessed whether the participant’s level of alcohol use could be considered problematic. Several studies have reported on the reliability of the AUDIT (Hays et al. 1995; Bohn et al. 1995) and results indicate high internal consistency (in Piccinelli et al. 1997).

The Traumatic Screening Questionnaire (TSQ; Brewin et al. 2002) screened patients for current symptoms of PTSD. The recommended cut-off score of six positive responses was adopted, which has a high overall prediction efficiency (0.90).

#### Experimental Session Measures

The Brief Symptom Inventory (BSI; Derogatis 1992) was an indicator of general psychiatric symptomatology and depression. Analyses focused on each of the nine symptom dimensions and the Global Severity Index (GSI). The test-retest reliability of GSI is high (0.84) with the symptom dimensions ranging between (0.68–0.91) (Derogatis 1992).

The Maudsley Addiction Profile (MAP; Marsden et al. 1998) assessed a participant’s substance use behaviour. The MAP is a self-report questionnaire comprised of five sections (A-E). Section A- management and demographic information; Section B- drug type, substance use in past 30 days including: frequency, amount, route of administration; Section C- health risk behaviours; Section D- physical health and psychological health problems and Section E-personal and social functioning. Sections D and E were omitted from the present study in order to reduce item measures. Across substances, inter-rater reliability (averaging 0.84) and test-retest reliability is high (averaging 0.88–0.94).

The Posttraumatic Stress Diagnostic Scale (PDS; Foa et al. 1997) assessed presenting symptoms of PTSD. The PDS has demonstrated good internal consistency (0.78–0.92), good test-retest reliability (0.77–0.81) and convergent validity with the structured clinical interview for PTSD diagnosis (0.65) and IES-R (0.78) (Foa et al. 1997; Foa and Riggs 1993)

The Emotional Processing Scale (EPS-25; Baker et al. 2007a) assessed different types of emotional processing styles. The EPS-25 contains 25 items and yields five factors of emotional processing. The EPS-25 measure has been found to have excellent internal consistency (0.88–0.92) and good test-retest reliability (0.74) (Baker et al. 2007b).

#### Emotional Processing Task

The computerised format of the International Affective Picture System (IAPS; Lang et al. 1997) assessed emotional processing. The IAPS is a set of normative emotional stimuli for experimental investigations of emotion and attention. The mean valence and arousal ratings for each picture have been published (Lang et al. 2008) and are highly internally consistent (0.94 and 0.93) and have excellent test-retest validity (0.99 and 0.97), (Lang et al. 2008). These ‘normed responses’ have yielded good agreement with control subjects in others studies (e.g. Spahic-Mihajlovic et al. 2005) and were deemed appropriate for use with the present population.

#### Selection of IAPS Pictures

Two sets of pictures were selected. The IAPS images were categorised a priori according to their pleasantness (i.e. unpleasant, neutral and pleasant) and intensity (i.e. low, medium, or high) based on published norms (Lang et al. 2008). The first set comprised ‘Set A’ of Lang’s original description of the ‘Looking at pictures test’ (Lang et al. 1993). A second set of 21 photographs ‘Set B’ were selected as previous studies have recommended using a larger number of IAPS images as well as trauma-related images to increase the sensitivity of the emotional measurement (Wolf et al. 2009). The IAPS tool enables flexibility to select additional picture sets, as there is a normative value for each of the individual photographs within the IAPS library.

‘Set B’ comprised included the same number and ratio of pleasant, unpleasant and neutral images followed the same randomisation methodology as ‘Set A’. They are listed here from the most pleasant (A) to unpleasant (U), with their IAPS number and actual order of presentation in parentheses: A (1750, 39); B (2071, 30); C (5829, 24); D (2299, 42); E (2373, 22); F (5726, 32); G (7001, 27); H (7002, 36); I (2695, 33); J (2745.2); K (2694, 34); L (2716, 31); M (2722, 41); N (2753, 25); O (2691, 29); P (2717, 40); Q (9903, 28); R (6313, 38); S (2345.1, 23); T (9220, 26); U (2703, 37). The order of both sets of photos, were randomised. However, ‘Set A’ always preceded ‘Set B’ in order to ensure consistency with previous studies that have employed ‘Set A’ published by Lang et al. (2008).

### Procedure

Participants were telephoned for a screening interview, unless they had requested to conduct the interview in person. A semi-structured interview was conducted to collect basic demographic information and included four screening questions related to drug use and experience of trauma. Depending on responses to items regarding previous traumatic experiences or substance use, potential participants were asked to complete one or more of the three screening questionnaires to assess current presenting symptoms and ensure suitability for the study.

The experimental sessions took place at either an addiction or trauma service site. Participants were interviewed in a quiet, private clinical room. Participants were then seated in front of a 14-in. (35 cm) computer monitor with the chair located approximately 2 ft. (0.6 m) from the screen to complete the computerised format of the IAPS (Lang et al. 1997). Task instructions adapted from the IAPS technical manual (Lang et al. 2008) were presented on the computer screen using Microsoft PowerPoint (2007) and were read out for those without English literacy. Participants then viewed and rated their emotional responses to two sets of 21 photographs, selected from IAPS. Each image was presented for 6 s. Following each presentation, participants were given 10-s to record their emotional response in an answer booklet on dimensions of “valence” and “arousal” using a 9-point self-assessment manikin (SAM) rating scale (Bradley and Lang 1994). A 4-s warning slide followed in order to prepare participants for viewing the next slide, which was then presented immediately. Participants were debriefed by the research investigator and given £10 compensation towards their time and travel costs. Participants were contacted within a week of completing the experimental session, in order to ensure that they were not experiencing any adverse effects (or intrusions) related to the photographic images.

## Results

### Demographics

Demographic data (Table 1) was analysed by one-way ANOVA, revealing that the groups were significantly different in ethnicity (F(2,83) = 6.37, *p* = 0.003), as there were a higher number of ethnic minorities within the PTSD-group. This difference was anticipated since a large number of participants for the PTSD-group, were refugees. There were a higher percentage of male participants recruited across the study as a whole (61.4%; *N* = 51), but no significant gender difference between groups.

**Table 1:** Sample demographics

Demographics	SUD-group (*N* = 26)	PTSD-group (*N* = 21)	SUD-PTSD group (*N* = 36)
Mean age (SD)	45.65 (14.24)	38.86 (11.93)	38.86 (7.81)
Sex (%)			
Male	20 (76.9)	12 (57.1)	19 (52.8)
Female	6 (23.1)	9 (42.9)	17 (47.2)
Ethnicity (%)			
White british	19 (73.1)	6 (28.6)	23 (63.9)
White other	3 (11.5)	5 (23.8)	5 (13.9)
Asian	-	2 (9.5)	-
Black caribbean	3 (11.5)	-	5 (13.9)
Black african	1 (3.8)	5 (23.8)	2 (5.6)
Other	-	3 (14.3)	1 (2.8)

### PTSD Symptom Severity

All participants within the PTSD and SUD-PTSD-group scored above clinical threshold on the TSC and PDS measures. Table 2 presents the median and quartiles for the three groups on the PDS. There was a significant difference between groups on the PDS total scores (H(2) = 38.44, *p* < 0.001), the number of trauma types (H(2) = 35.83, *p* < 0.001) and on all the PDS symptom dimensions: re-experiencing (H(2) = 41.57), avoidance (H(2) = 35.83, *p* < 0.001) and arousal (H(2) = 35.04, *p* < 0.001) as well as on the impact of symptoms on daily functioning (H(2) = 34.98, *p* < 0.001). Pair-wise comparisons indicated the SUD-group scored significantly lower than both PTSD and SUD-PTSD groups on the PDS total and all PDS symptom dimensions (Mann-Whitney U test; all *p* < 0.0001). In addition, the PDS total (*p* = 0.024), number of trauma types (*p* = 0.004) and intrusive symptoms (*p* = 0.001) were significantly higher in the PTSD-group compared to the SUD-PTSD-group. However, these two groups did not differ on avoidance and arousal symptoms or on the impact of symptoms on daily functioning.

**Table 2:** Exposure to Trauma and PTSD symptom severity

PTSD symptom severity (PDS)	SUD-group (*N* = 15) Median (lower- upper quartiles)	PTSD-group (*N* = 21) Median (lower- upper quartiles)	SUD-PTSD group (*N* = 36) Median (lower- upper quartiles)
Mean no. reported traumas (SD)	1.3 (1.28)	5.61 (2.36)	3.8 (2.23)
Symptom dimensions			
Re-experiencing symptoms	0.0 (0–0)	13.0 (10.5–13)	8.5 (5–12)
Avoidance symptoms	0.0 (0–0)	15 (10–17.5)	13 (8–17)
Arousal symptoms	0.0 (0–2)	10 (8–12.5)	10 (8–13)
No. of areas of daily functioning affected (PDS)	0.0 (0–0)	7.0 (5.5–7.5)	7.0 (5–9)
Total PDS symptom severity	0 (0–3.0)	38.00 (31.5–42.0)	30.50 (26.0–39.0)

### Substance Use Severity

All participants in the SUD and SUD-PTSD groups scored above clinical threshold on either or both the SDQ, AUDIT and MAP measures. Table 3 presents the monthly mean usage of substances by group and monthly risk behaviour symptoms. Across both the SUD-PTSD and SUD groups (*N* = 62) the majority of participants reported using more than one substance (*N* = 57; 91%) and reported higher levels of depressant as opposed to stimulant substance use. SUD and SUD-PTSD groups did not differ significantly on each substance for monthly substance use or for risk behaviours symptom severity.

**Table 3:** Group means on the MAP

MAP Items (B-D)	SUD- group Mean (N)	PTSD-group Mean (N)	SUD-PTSD group Mean (N)
B. Substance use in last month			
Alcohol mean (units)	261.26 (*N* = 21)	2.38 (*N* = 3)	129.49 (*N* = 24)
Heroin (grams)	7.75 (*N* = 5)	-	5.1 (*N* = 9)
Crack (grams)	24.18 (*N* = 7)	-	29.08 (*N* = 14)
Cocaine (grams)	0.0385 (*N* = 1)	-	0.68 (*N* = 3)
Cannabis (oz)	1.48 (*N* = 11)	-	2.94 (*N* = 14)
Prescribed & Illicit benzodiazepines (tablets)	120.19 (*N* = 7)	-	100.83 (*N* = 8)
Prescribed methadone (mls)	1153.3 (*N* = 12)	-	1785.48 (*N* = 23)
Prescribed diamorphine (mls)	4628.57 (*N* = 4)	-	2000 (*N* = 2)
Other	(*N* = 2)	-	(*N* = 3)
C. Risk behaviour in last month			
IV use	10.2 (*N* = 8)	-	18.1 (*N* = 6)
Shared IV use	0.12 (*N* = 1)	-	0.25 (*N* = 1)
Sex with more than one sexual partner	0.42 (*N* = 1)	0.06 (*N* = 0)	0.39 (*N* = 1)
Unprotected sex	0.1 (*N* = 2)	0.00 (*N* = 0)	0.52 (*N* = 4)

### Psychiatric Symptom Severity

One-way ANOVA revealed a significant difference between groups on the BSI total score (F(2,73) = 7.03, *p* = 0.002) and on seven out of nine subscale scores: obsessive compulsive (F(2,73) = 4.09, *p* = 0.021); interpersonal sensitivity (F(2,72) = 6.31, *p* = 0.003); depression (F(2,73) = 4.153, *p* = 0.02); anxiety (F(2,73) = 5.53, *p* = 0.006); hostility (F(2,73) = 3.48, *p* = 0.036); phobic anxiety (F(2,73) = 9.71, *p* < 0.001); psychoticism (F(2,73) = 5.23, *p* = 0.008). There was also a significant group difference on the additional items (F(2,73) = 4.15, *p* = 0.002). Both PTSD and SUD-PTSD groups scored higher on levels of psychiatric symptom severity demonstrated by the total BSI and its symptom dimensions other than paranoia and somatisation, (post hoc LSD-test; *p* < 0.05). There were no significant differences between the SUD-PTSD and PTSD groups on psychiatric symptom severity.

### Emotional Processing

Table 4 presents the group means and standard deviations for the EPS-25 total and the five dimensions of emotional processing. The dimension means were calculated by dividing the group means by the number of questionnaire items within each dimension. Dimension means for all three groups fell within clinical range for all five-symptom dimensions, when compared to normative EPS-25 values.

**Table 4:** Severity of emotional processing abnormality as measured by the EPS-25 and Dimension Mean totals

EPS-25 dimensions	SUD-group Mean (SD) Dimension Mean	PTSD-group Mean (SD) Dimension Mean	SUD-PTSD group Mean (SD) Dimension Mean
Suppression	23.8 (12.9)	28.15 (7.97)	30.15 (10.46)
	4.7	5.7	6.03
Unprocessed	24.1 (11.46)	31.4 (7.88)	30.71 (7.75)
	4.82	6.28	6.14
Unregulated	16.7 (10.92)	21.85 (8.29)	25.13 (7.4)
	3.34	4.37	4.19
Avoidance	20.94 (9.74)	26.63 (10.71)	27.06 (8.57)
	4.19	5.33	5.41
Impoverished	22.0 (8.43)	26.15 (9.47)	26.21 (8.85)
	4.4	5.23	5.15
EPS-25 mean total	101.40 (41.4)	128.78 (33.34)	133.16 (28.21)
Dimension score	4.056	5.15	5.33

There were significant differences between groups on the EPS-25 total scores (one-way ANOVA; F(2,75) = 6.43, *p* < 0.003), as well as on emotional processing dimensions of: unprocessed emotion (F(2,75) = 4.76, *p* < 0.01), unregulated emotion (F(2,75) = 6.18, *p* < 0.003) and avoidance (F(2,75) = 3.27, *p* < 0.04). Post-hoc LSD-test indicated a significant difference between the SUD-group and both the SUD-PTSD (*p* = 0.001) and PTSD groups (*p* = 0.01) on the EPS-25 total scores. Both PTSD and SUD-PTSD groups displayed significantly more emotional processing difficulties on the EPS-25 than the SUD-group. The SUD-group was also significantly less impaired than both groups on the emotional processing dimensions of unprocessed emotion (*p* < 0.01) and significantly less impaired than the SUD-PTSD-group (only) on dimensions of suppression (*p* = 0.032), unregulated emotion (*p* = 0.001) and avoidance (*p* = 0.02). There were no significant differences between the SUD-PTSD and PTSD groups on the EPS-25 total or symptom dimensions.

Group mean valence and arousal responses to pleasant and unpleasant pictures are presented in Table 5 with normative values for the IAPS pictures.

**Table 5:** Severity of emotional processing abnormality as measured by the IAPS

IAPS Data	Normative values	SUD-group	PTSD-group	SUD-PTSD group
		Mean (SD)	Mean (SD)	Mean (SD)
Valence				
Pleasant pictures	115.36	111.44 (19.16)	105.86 (14.48)	100.37 (15.68)
Unpleasant	65.33	59.83 (18.42)	54.86 (16.49)	67.71 (18.12)
Arousal				
Pleasant	76.22	71.52 (38.27)	67.95 (36.13))	50.17 (28.36)
Unpleasant	118.83	95.72 (41.28)	116.48 (34.89)	92.75 (35.08)

**Fig. 1:**
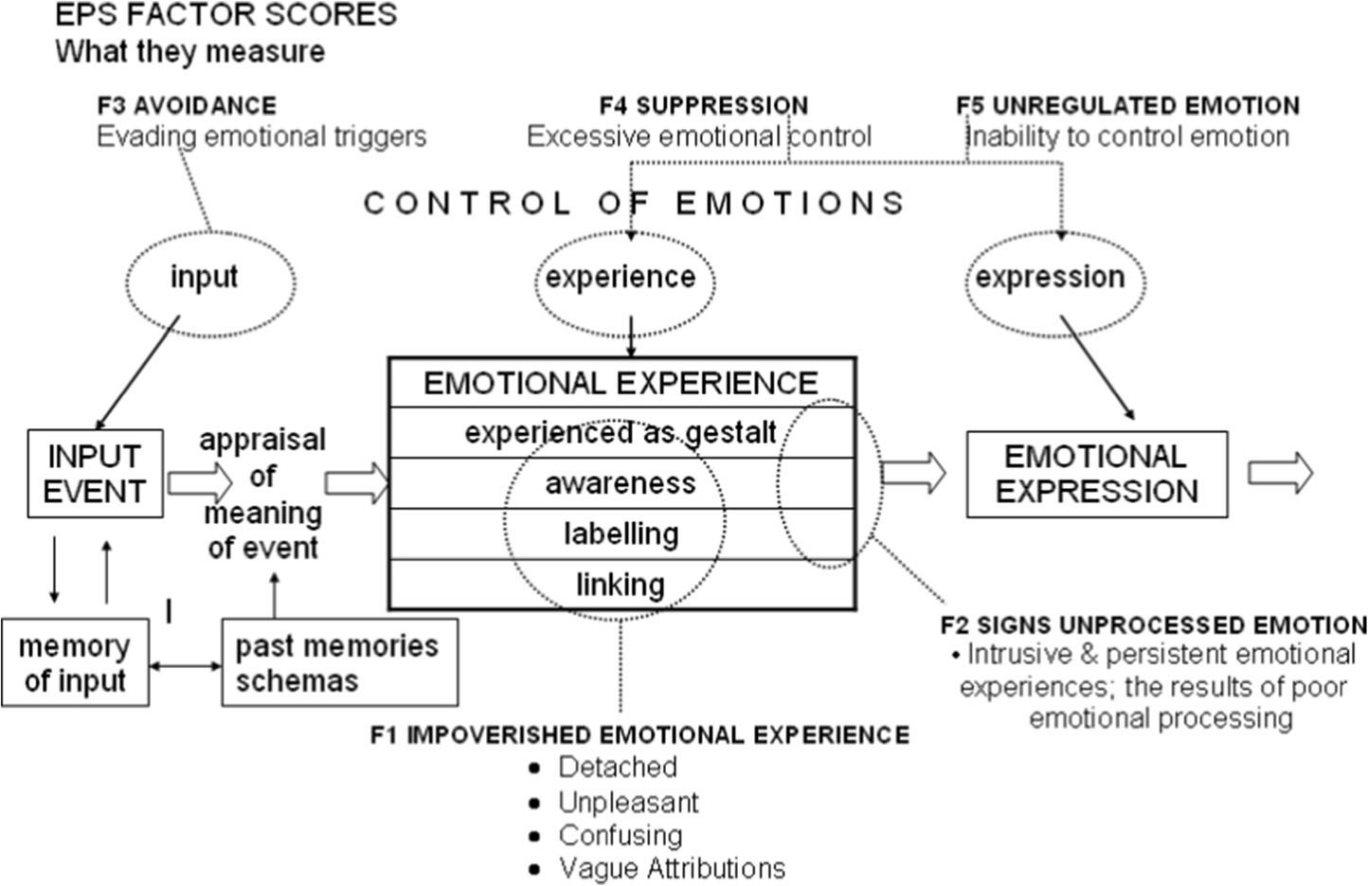
Model of main domains of emotional processing, Baker et al. (2007b)

#### Valence

**Fig. 2:**
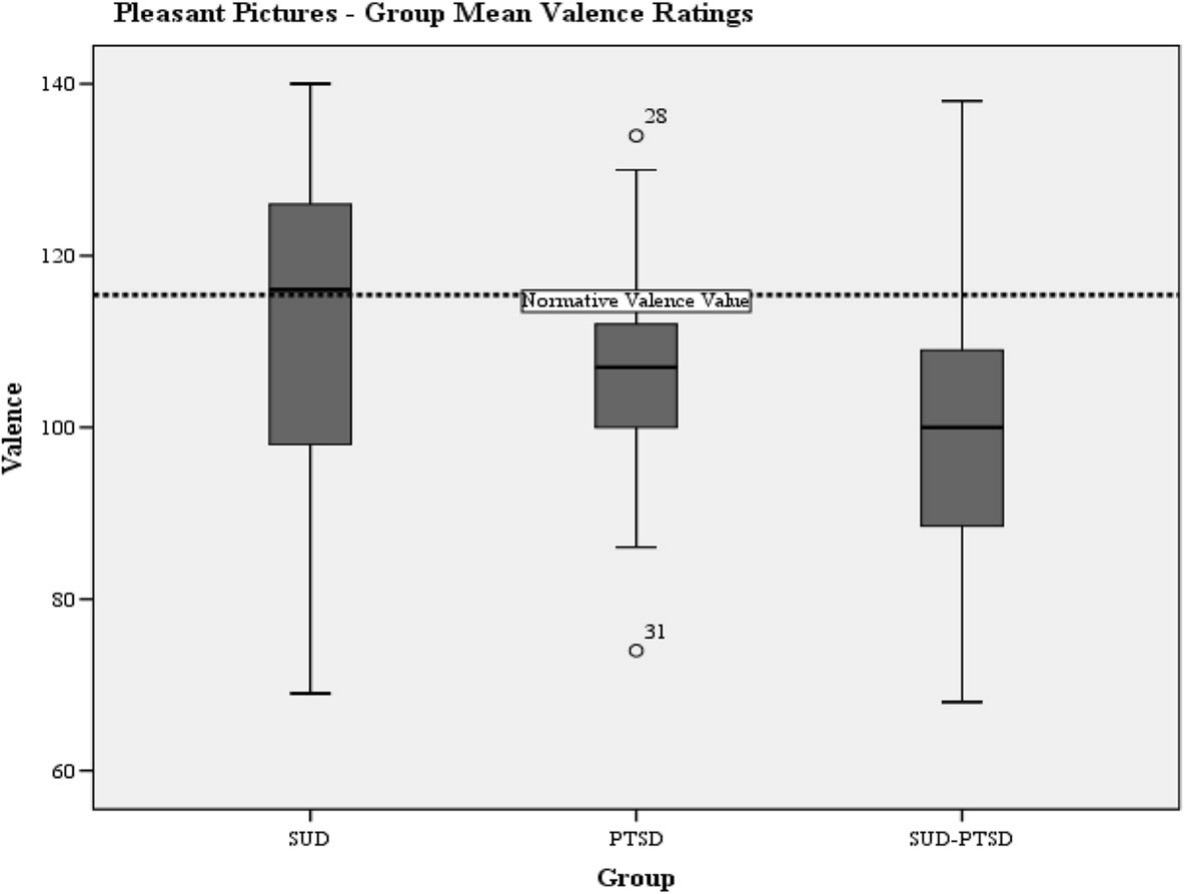
Boxplots of the group mean valence ratings to pleasant pictures. The horizontal dotted lines indicate the normative IAPS mean valence values for pleasant pictures. Two outliers are depicted within the PTSD group valence responses to pleasant pictures

**Fig. 3:**
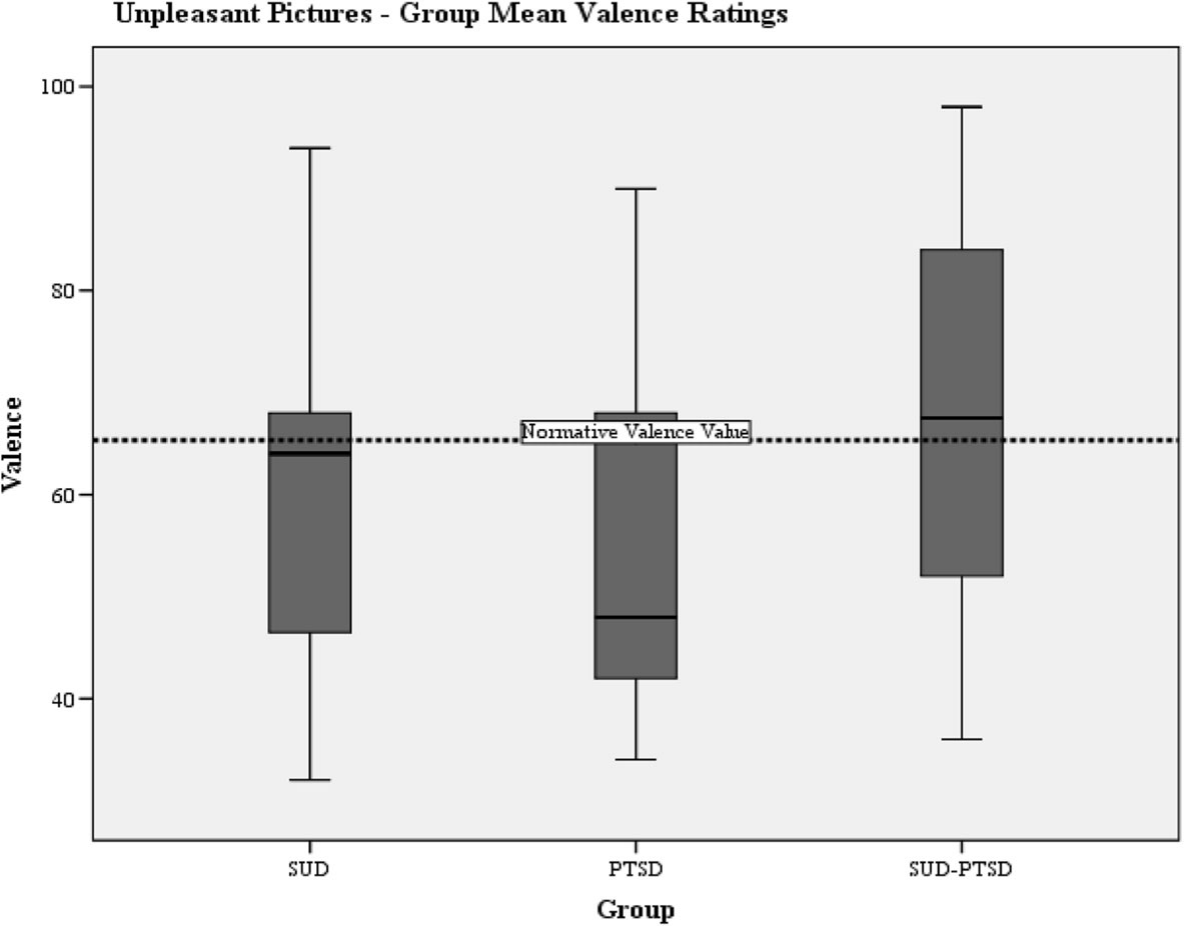
Boxplots of the group mean valence ratings to unpleasant pictures. The horizontal dotted lines indicate the normative IAPS mean valence values for unpleasant pictures

One-way ANOVA indicated a significant difference of valence between groups for pleasant pictures (F(2,80) = 3.29, *p* = 0.043) and unpleasant pictures (F(2.78) = 3.62, *p* = 0.032). The SUD-PTSD-group reported lower mean valence to pleasant pictures than the SUD-group (post hoc LSD-test; *p* = 0.013), but the SUD-PTSD and PTSD group did not differ on this measure (Fig. 2). There was also a significant group difference between the SUD-PTSD-group and the PTSD-group to unpleasant pictures on valence whereby, the PTSD-group reported lower valence to unpleasant pictures than the SUD-PTSD-group (Fig. 3). T-tests were performed to see if there were any differences between groups to the normative valence values. Both the SUD-PTSD and PTSD groups scored significantly lower that the norm valence value for pleasant pictures: PTSD-group (*t*(2,20) = −3.01, *p* = 0.007) and the SUD-PTSD-group (*t*(2,34) = −5.65, *p* < 0.001), which suggests that both groups found the pictures less pleasant than the normal population. The PTSD-group scored significantly lower than the norm valence mean value to unpleasant pictures (*t*(2,20) = −2.91, *p* < 0.009), which suggests that the PTSD-group found unpleasant pictures even less pleasant than the normal population.

#### Arousal

**Fig. 4:**
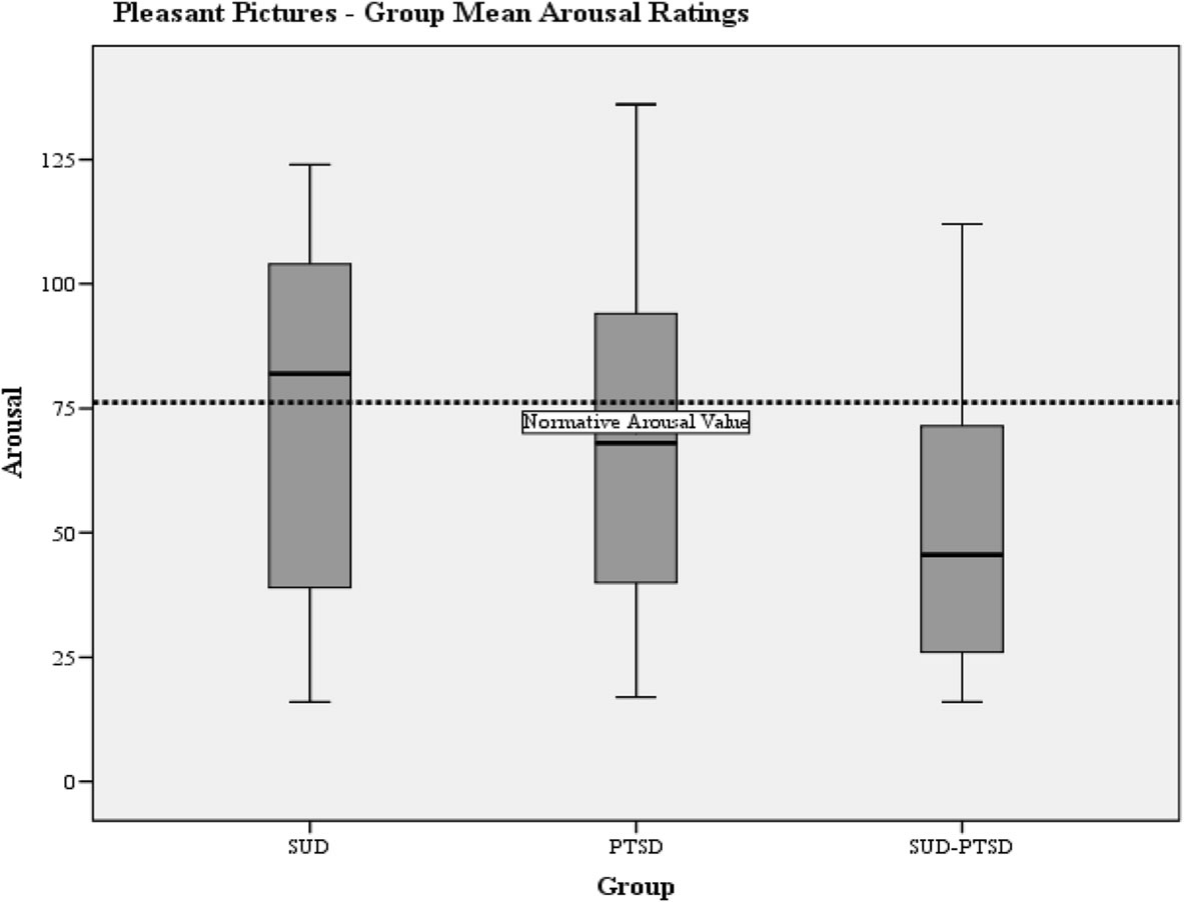
Boxplots of the group mean arousal ratings for pleasant pictures. The horizontal dotted lines indicate the normative IAPS mean arousal values for pleasant pictures

**Fig. 5:**
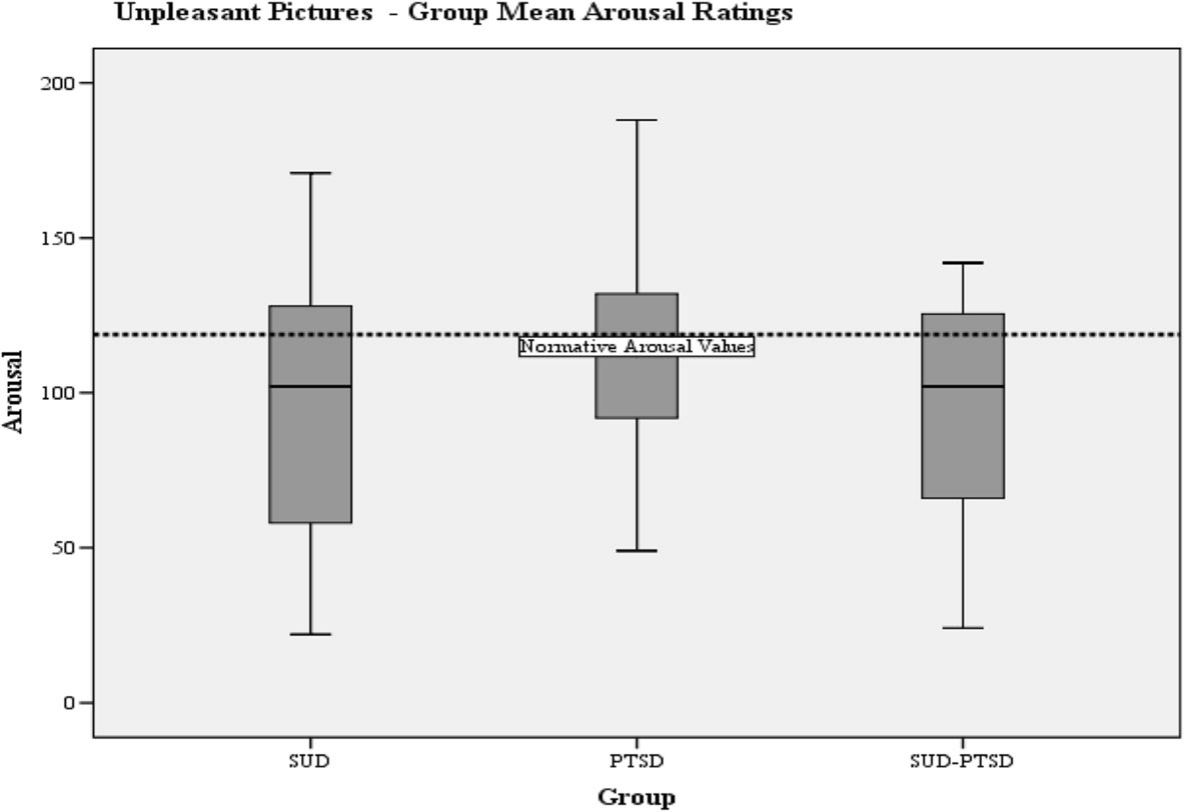
Boxplots of the group mean arousal ratings for unpleasant pictures. The horizontal dotted lines indicate the normative IAPS mean arousal values for unpleasant pictures

One-way ANOVA indicated a significant group difference of arousal to pleasant pictures (F(2.81) = 3.54, *p* = 0.034). The SUD-PTSD group reported significantly lower levels of arousal to pleasant pictures than the SUD-group (LSD-test; *p* = 0.017) (Fig. 4). One-way ANOVA indicated a trend towards a group difference for arousal to unpleasant pictures (F (2,81) = 2.934, *p* = 0.059) (Fig. 5).

T-tests were performed to see if there were any differences between the groups to the normative arousal values to pictures. The SUD-PTSD group scored significantly lower that the normative arousal value to pleasant pictures: SUD-PTSD-group (*t*(2,35) = −5.51, *p* < 0.001), which indicates that the SUD-PTSD found pleasant pictures less arousing than the normal population. Both the SUD-PTSD and SUD-groups scored significantly lower that the norm arousal value to unpleasant pictures: SUD-PTSD-group (*t*(2,35) = −4.46, *p* < 0.001) and SUD-group (*t =* (2, 24) = − 2.80, *p* = 0.01). There was no significant difference between the PTSD group and the normative arousal value to unpleasant pictures.

## Discussion

In line with previous findings, patients with co-morbid SUD-PTSD report higher levels of psychiatric symptomatology than patients with SUD only (e.g. Brown and Ouimette 1999). The PTSD-group had higher overall symptom severity scores and higher levels of intrusive PTSD symptoms. There were however, no significant group differences between the PTSD and SUD-PTSD groups on symptoms of avoidance, and arousal or on the impact of PTSD symptoms on daily functioning.

All three groups exhibited evidence of emotional processing dysfunction, scoring within the clinical range on the EPS-25 total score and for all five dimensions of emotional processing abnormalities. The SUD-PTSD and PTSD groups scores indicate significantly more emotional processing difficulties than the SUD-group on the EPS-25 total and on the emotional processing dimensions of unprocessed emotion, unregulated emotion and avoidance, which may map onto the PTSD symptom dimensions of ‘re-experiencing’, ‘emotional numbing’ and ‘avoidance’. All three groups also scored lower on levels of valence and arousal in response to pleasant pictures than the normal population. Both the SUD-PTSD and PTSD groups scored significantly lower on levels of valence and arousal in response to pleasant pictures than the SUD-group and the normal population, which suggests evidence of emotional numbing and positive blunting.

In relation to the groups’ responses to unpleasant pictures the findings were more complex. There was some evidence of heightened negativity in response to unpleasant stimuli as the PTSD group reported significantly lower valence to unpleasant pictures than the normative valence values as well as the SUD-PTSD group. However, both the SUD-PTSD and SUD groups scored significantly lower on arousal scores to unpleasant pictures than the normal population, which may be interpreted as evidence of emotional numbing and negative blunting.

These findings are contrary to the expectations based on previous research that has reported substance use heightened negativity (Verdejo-García et al. 2006). However, the present results accord with findings from studies that have reported a tendency toward neutral valuations (i.e. reduced valence to both pleasant and unpleasant pictures and reduced arousal to all stimuli) in depressant substance users (Aguilar de Arcos et al. 2005). Findings of increased valence and reduced arousal responses to unpleasant stimuli are not necessarily surprising. These findings may align with the self-medication hypothesis and the view that some individuals with SUD report using substances to help block, suppress or numb painful trauma memories (e.g. Reynolds et al. 2004). In contrast to previous suggestions, therefore, substance use did not appear to heighten the effect of unpleasant stimuli in trauma, which appears to be one of the primary reasons for withholding trauma-focused treatment. There were also no significant differences between the SUD-PTSD and SUD groups to the normative valence values for unpleasant pictures, which suggests that both of these groups are still (to a certain degree) able to process the emotive response of unpleasant stimuli. This is also in line with a recent study examining IAPS responses in heavy cannabis users that reported users did not judge fewer stimuli as emotional compared to controls (Wesley et al. 2016).

One limitation of the study was that a proportion of those recruited from the trauma services were refugee clients. This may have resulted in the significant differences between the PTSD and SUD-PTSD groups in relation to trauma type and symptom severity, as a high number of participants in the PTSD-group were survivors of torture. Conclusions in relation to group differences between the SUD-PTSD and PTSD groups may therefore have been confounded by group differences in trauma type and trauma symptom severity. Linked to the limitations relating to having a higher numbers of refugees in the trauma group, there was also a significant difference between groups in relation to ethnicity. This meant that there were also a higher number of participants in the PTSD-group who spoke English as a second language. In order to limit any effects of possible language barriers, the research team were careful to only select participants who were fluent in spoken English. This was facilitated through liaison with the referring clinicians and through the screening interview. The research team also read out questionnaire items and instructions where necessary.

A further limitation of the present study was that the majority of participants were poly-substance and alcohol users. Due to the sample size, it wasn’t possible to explore emotional processing dysfunction by substance type due to the small numbers in each of the substance type categories and the overlap between substances. Although this was a representative sample of service users presenting to drug and alcohol services, there may be differences in emotional processing between substance users that weren’t detected due to poly substance and alcohol use. Another consideration is that although participants were requested to refrain from alcohol or illicit substances for at least 24 h prior to assessment they were not asked to provide a urine sample for screening before taking part in the assessment and therefore it is possible that some of the participants may have been under the influence of substances at time of assessment. Seven participants were included for various reasons including evident intoxication during the assessment however, it is possible that some participants may have been under the influence of substances did not disclose their substance use or present with evident intoxication. Some participants were also receiving prescribed medication including methadone which again is representative of the sample however may have impacted results and would warrant further investigation. A larger sample size would facilitate further grouping of participants by substance type as well as prescribed medication such as those on methadone maintenance treatment.

Another key consideration is the sensitivity of the IAPS tool for measuring emotional processing. Researchers have questioned the sensitivity of the IAPS rating protocol for the assessment of PTSD-related emotional numbing and recommendations have been made to increase the sensitivity of the assessment tool including, increasing the number of pictures and including trauma-related images (Wolf et al. 2009). For this reason a second set of 21 photographs ‘Set B’ were selected as previous studies have recommended using a larger number of IAPS images as well as trauma-related images to increase the sensitivity of the emotional measurement (Wolf et al. 2009). However, a recent study using IAPS to assess emotional responses in heavy cannabis users found that cannabis users did not judge fewer stimuli on the IAPS task as emotional compared to controls but did report abnormal medial prefrontal cortex activity. This suggests that there may be some underlying abnormal activity at a neurobiological level that the IAPS task is not sensitive enough to assess. That said this study found that not all activity patterns in cannabis users were abnormal during conscious emotional evaluation and taken together, their findings suggest that *“many affect-related processes appear normal in long-term heavy cannabis users who are not intoxicated during conscious emotional evaluation”* (Wesley et al. 2016, p. 1039).

Despite the above limitations, these findings add to the literature within both the trauma and addiction fields, which implicates the role of impaired emotional processing in patients with co-morbid SUD-PTSD. Overall both the SUD-PTSD and PTSD groups appear to be equally affected by the impact of PTSD symptoms on their daily living and psychiatric symptom severity; they also presented with similar emotional processing difficulties. Within the UK, the evidence-based treatment for PTSD is trauma-focused cognitive-behavioural treatment (CBT) or eye movement desensitisation reprocessing (EMDR) which involves prolonged exposure (PE). However, UK guidelines recommend that patients with dual diagnosis (SUD-PTSD) should not be offered trauma-focused treatment until they are substance free (NICE 2005) which contradicts the Department of Health “best practice” guidelines which recommend an integrated treatment model (DOH 2002).

In conclusion, each of the three groups reported evidence of emotional processing dysfunction relative to the normal population. Within the SUD-PTSD group there was significant evidence that the additional impact of trauma increased emotional processing dysfunction but less evidence to suggest that substance use increased emotional processing dysfunction or heightened negative responses. As discussed, integrated treatment for SUD-PTSD including trauma-focused therapies with exposure, remain largely underutilised with SUD-PTSD patients (Becker et al. 2004). Anticipated emotional processing impairments as well as fears around safety and exacerbating symptoms appear to be the central barriers for offering exposure-based treatment for patients suffering with SUD-PTSD (e.g. Ehlers and Steil 1995). Given that the present findings show relatively little difference between the SUD-PTSD and the PTSD groups on emotional processing dysfunction, doubts are raised about the appropriateness of UK treatment guidelines for patients presenting with co-morbid SUD-PTSD (NICE 2005) as well as the on-going reluctance to incorporate exposure based techniques in the care of individuals presenting with co-morbid SUD-PTSD (Gulliver and Steffen 2010). Future research directions would benefit from an integrated treatment trial for co-morbid SUD-PTSD, which includes the assessment of emotional processing difficulties whilst individuals are involved in exposure-based therapies. This would determine with greater certainty what emotional processing difficulties arise for these three client groups and to evaluate whether they interfere with the effectiveness of trauma-focused treatments.
